# TGFβ depletion does neither modulate acute E. coli-induced inflammatory immune responses nor impair the protective effect by chronic filarial infection

**DOI:** 10.3205/id000044

**Published:** 2019-11-12

**Authors:** Benedikt C. Buerfent, Jesuthas Ajendra, Wiebke Stamminger, Fabian Gondorf, Achim Hoerauf, Marc P. Hübner

**Affiliations:** 1 Institute of Medical Microbiology, Immunology and Parasitology, University Hospital Bonn, Germany; 2 Center for Human Genetics, University Hospital of Marburg, Germany; 3 Lydia Becker Institute for Immunology & Infection, Faculty of Biology, Medicine & Health, Manchester Academic Health Science Centre, University of Manchester, United Kingdom; 4 Life and Medical Sciences (LIMES) Institute, Immunology & Environment, University of Bonn, Germany; 5 German Center for Infection Research (DZIF), partner site Bonn-Cologne, Bonn, Germany

**Keywords:** TGF beta, sepsis, E. coli, immune modulation, systemic pro-inflammatory response syndrome (SIRS), Litomosoides sigmodontis, helminth, filaria, nematode

## Abstract

TGFβ is an anti-inflammatory molecule that suppresses pro-inflammatory immune responses. Previously, we demonstrated that chronic filarial infection has a beneficial impact on *Escherichia coli*-induced sepsis. In the present study, we investigated whether this protective effect is dependent on TGFβ signaling and whether depletion of TGFβ before *E. coli* challenge alters the early course of sepsis *per se*. *In vivo* depletion of TGFβ before *E. coli* challenge did not alter levels of pro-inflammatory cytokines/chemokines and did neither increase the bacterial burden nor worsen *E. coli*-induced hypothermia six hours post *E. coli* challenge. Similarly, in the co-infection model, despite TGFβ depletion, mice infected with the filarial nematode *Litomosoides* *sigmodontis* exhibited milder *E. coli*-induced hypothermia, reduced bacterial load and pro-inflammatory immune responses. Thus, we conclude that TGFβ is not essentially modulating the initial pro-inflammatory phase during sepsis and that the protective effect of a chronic filarial infection against sepsis is independent of TGFβ signaling.

## Introduction

TGFβ terms an anti-inflammatory cytokine superfamily including the isoforms TGFβ 1–3, which are produced by several immune cell types [1]. TGFβ has important regulatory functions, inhibits cell proliferation, triggers wound healing and has implications in cancer and autoimmune diseases [1], [2]. During helminth infection, a regulatory milieu is induced, which allows long-term survival of the parasites in the host and influences bystander immune responses. Protection against type 1 diabetes onset in nonobese diabetic mice infected with the filarial nematode *Litomosoides sigmodontis* required TGFβ [3]. Similarly, depletion of TGFβ reversed the *L. sigmodontis*-mediated amelioration of airway hyperreactivity in a murine asthma model [4]. 

Several studies implicated that TGFβ may also be involved during the course of sepsis [5], [6], [7]. Lack of TGFβ 1 was associated with an increased TLR4 expression and resulted in LPS hyperresponsiveness with increased levels of nitric oxide and inflammatory cytokines [8]. Consequently, TGFβ pre-treatment reduced nitric oxide release and improved LPS-induced endotoxemia in rats [9]. Recently, we demonstrated that chronic *L. sigmodontis* infection protected mice against *Escherichia coli*-induced sepsis via TLR2-dependent macrophage modulation, resulting in a milder *E. coli*-induced hypothermia, reduced inflammation, improved bacterial clearance and sepsis survival [10]. Furthermore, *L. sigmodontis* infection did not exacerbate the immune paralysis during the late phase of sepsis [11]. *Brugia malayi* filarial extract also modulates LPS-induced immune responses in human monocytes and induces pentraxin-3 (PTX3) expression, a soluble pathogen recognition receptor, and expression of CXCL5 and CXCL6 [12]. In the present study, we investigated the role of TGFβ during the course of an *E. coli* challenge and whether TGFβ is essential for the protective effect seen in *L. sigmodontis*-infected mice.

## Methods

### Ethics statement and mice 

Female BALB/cJ mice (Janvier, Saint-Berthevin, France) were maintained at the Institute for Medical Microbiology, Immunology and Parasitology of the University Hospital Bonn, Germany. All experiments were performed according to the European Union animal welfare guidelines and approved by the Landesamt für Natur, Umwelt und Verbraucherschutz, Cologne, Germany (AZ 84-02.04.2011.A326).

### Filarial infection model and in vivo depletion of TGFβ

Mice were infected with *L. sigmodontis* at 6–8 weeks of age via the tropical rat mite *Ornithonyssus bacoti* as previously described [13]. Chronic filarial infection was confirmed by the presence of adult worms within the thoracic cavity at the time of necropsy. Systemic TGFβ 1–3 was depleted on day –3 and day –1 before *E. coli* challenge by intraperitoneal injection of 100 µg/mouse anti-TGFβ depletion antibody (Clone: 1D11.16.8, BioXCell, West Lebanon, USA), a dose which was previously shown to prevent the protection against diabetes onset by filarial infection in nonobese diabetic mice [3]. Controls received 100 µg/mouse IgG1 isotype control (Clone: MOPC-21, BioXCell). 

### Bacterial challenge and cell preparation 

Ninety-day-*L. sigmodontis*-infected mice and age-matched controls were intraperitoneally injected with 8.5x10^8^–1.0x10^9^ cfu of *E. coli* K12 (ATCC 25922) in 200 µL sterile LB medium and monitored for 6 h. Body temperature was taken hourly using an infra-red thermometer. After 6 h, blood was taken and mice were euthanized by an overdose of isoflurane (AbbVie, Wiesbaden, Germany). The peritoneal cavity was lavaged with 5 mL RPMI 1640 advanced medium (Gibco^®^). The first mL was used to quantify the bacteria on LB agar plates after overnight incubation at 37°C and to quantify cytokine/chemokine concentrations after centrifugation at 300 g for 10 min. The blood was centrifuged at 6,000 g for 5 min and both serum and first mL of peritoneal lavage were stored at –20°C. 

### Flow cytometry and enzyme linked immunosorbent assay 

**Figure 1:**
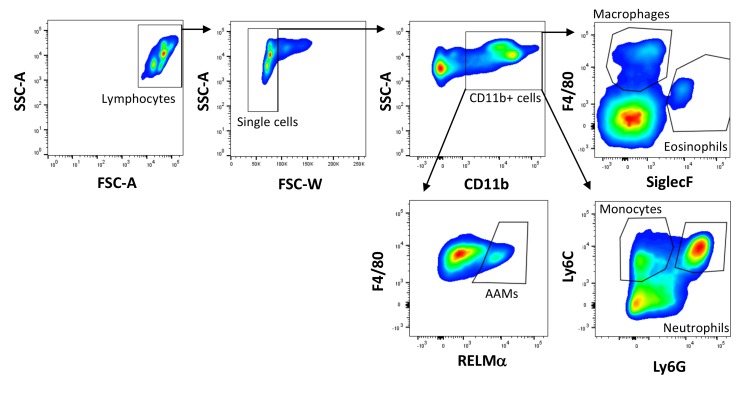
Gating strategy used for flow cytometry. Lymphocytes were gated by forward and side scatter and single cells were identified by a lower FSC-W. CD11b^+^ myeloid cells were gated based on CD11b positivity and macrophages were identified as CD11b^+^F4/80^+^SiglecF^–^, eosinophils as CD11b^+^F4/80^low^SiglecF^+^, monocytes as CD11b^+^Ly6C^+^Ly6G^–^ and neutrophils as CD11b^+^Ly6C^+^Ly6G^+^ cells. Alternatively activated macrophages were identified as RELMα positive based on the fluorescence minus one approach.

For flow cytometric analysis, cells were blocked with PBS containing 1% bovine serum albumin and 0.1% rat IgG (Sigma-Aldrich, St. Louis, USA) for 30 min. After a washing step, cells were stained with SiglecF-PE or -AlexaFluor647 (Clone: E50-2440, BD Pharmingen, San Diego, USA), F4/80-PerCP-Cy5.5 (Clone: BM8), Ly6G-PE (Clone: 1A8), Ly6C-APC-Cy7 (Clone HK1.4) (all BioLegend, San Diego, USA), and CD11b-FITC or -PE-Cy7 (Clone: M1/7; eBioscience, San Diego, USA). For intracellular staining, cells were incubated with fixation and permeabilization buffer (eBioscience) overnight. Cells were stained with rabbit anti-mouse RELMα (Peprotech, Rocky Hill, USA) followed by donkey anti-rabbit IgG AlexaFluor647 (Clone: Poly4064, BioLegend) and CD86-PE (Clone: GL1) and MHCII-APC (Clone: M5/114.15.2, eBioscience) to determine cell activation. The gating strategy used to identify macrophages, monocytes, eosinophils and neutrophils is shown in Figure 1 (Fig. 1).

IFNγ , IL-10, TGFβ and TNF (all eBioscience) as well as CXCL1/KC and CXCL2/MIP-2 (both R&D, Minneapolis, USA) were measured from peritoneal lavage and serum by ELISA according to the manufacturers’ protocols and analyzed using a plate reader (Molecular Devices) with SoftMax Pro 6.

### Data management and statistical analysis 

Flow cytometry data were generated using a BD FACS Canto and BD FACS Diva 6.0 software (BD Bioscience) and analyzed by FlowJo V10 software (Tree Star, Ashland, USA). The statistical analysis was performed using Prism GraphPad 5.01 (GraphPad Software, San Diego, USA). Normal distribution of data was tested with D’Agostino & Pearson test. Normally distributed data were tested for statistical significance using 1-way ANOVA with Tukey’s multiple comparisons test or 2-way ANOVA and Bonferroni post hoc test. Data that was not normally distributed was tested for statistical significance using Kruskal-Wallis test followed by Dunn’s multiple comparison post hoc test. Box and Whisker blots show minimum and maximum; bar graphs represent means +SEM. Associations were tested by Spearman’s rank correlation coefficient. 

## Results

### Filaria-mediated protective effects on E. coli-induced hypothermia, peritoneal bacterial burden, macrophage numbers and activation are unaffected by TGFβ depletion 

**Figure 2:**
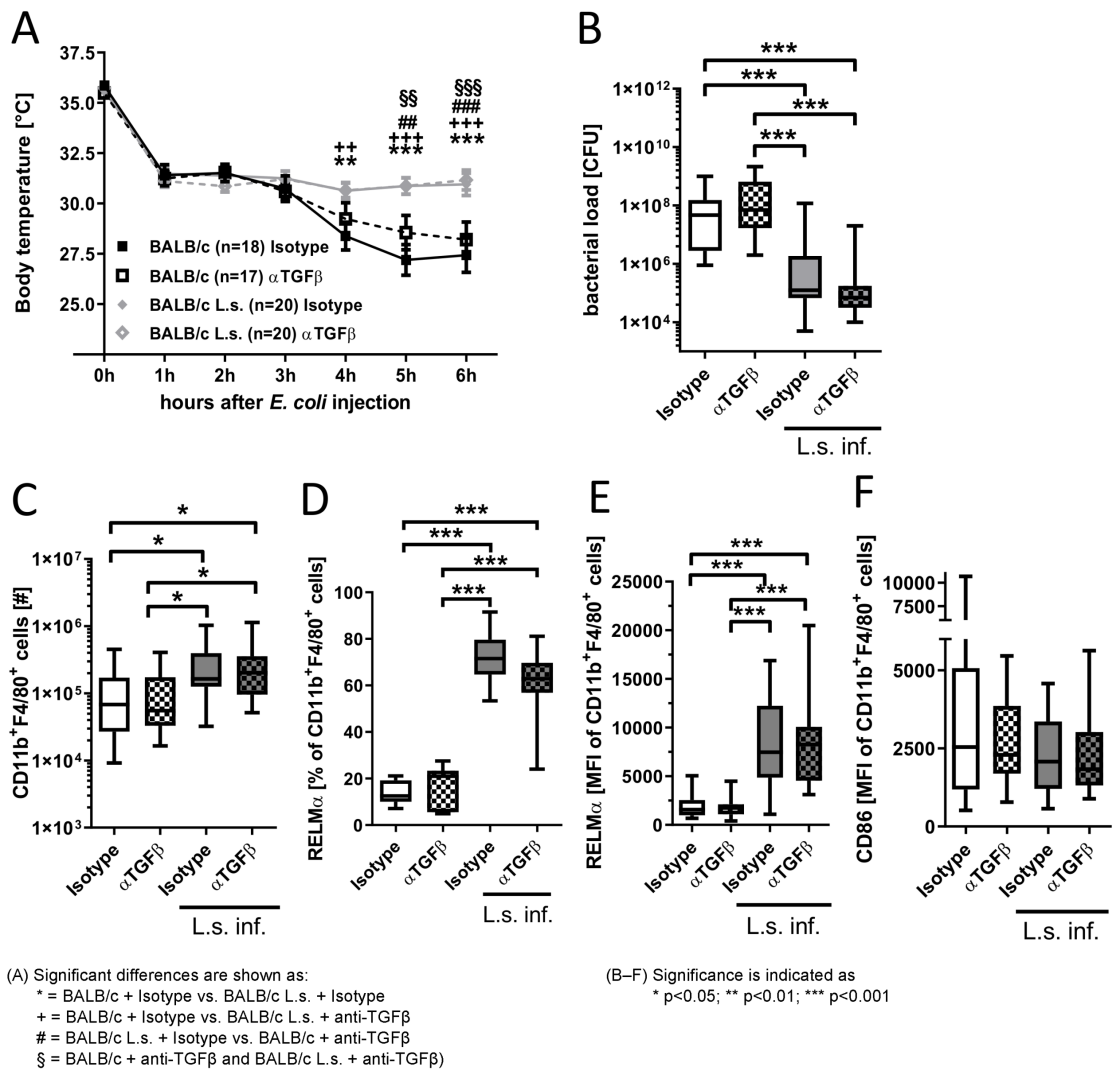
TGFβ depletion does neither alter *E. coli*-induced hypothermia, peritoneal bacterial burden, nor impair the filaria-mediated protective effect. (A) Kinetic of the body temperature after intraperitoneal injection of 8.5x10^8^–1.0x10^9^ cfu *E. coli* K12 in chronic *L. sigmodontis* (L.s.)-infected BALB/c mice (L.s. Isotype: n=20; L.s. anti-TGFβ: n=20) and uninfected controls that received either anti-TGFβ or an isotype control before *E. coli* challenge (Isotype: n=18; anti-TGFβ: n=17. (B) Peritoneal bacterial load [cfu], (C) absolute cell number of CD11b^+^F4/80^+^ macrophages as well as (D) the frequency of macrophages expressing RELMα and macrophage mean fluorescence intensity (MFI) of (E) RELMα and (F) CD86 six hours after *E. coli* injection. Pooled data of three independent experiments with at least 6 mice per group. Data is shown as median (A), box and whisker blots with min/max (B–F) and was tested for statistical significance by 2-way ANOVA and Bonferroni post hoc test (A), Kruskal-Wallis test and Dunn’s Multiple Comparison post hoc test (B,C,E–F) or 1-way ANOVA with Tukey’s multiple comparisons test (D).

To investigate whether TGFβ is implemented in the SIRS phase and the protective responses provided by *L. sigmodontis* infection, TGFβ was depleted before *E. coli* challenge in chronic *L. sigmodontis*-infected and uninfected mice and the impact on hypothermia and bacterial burden was assessed. Six hours after *E. coli* injection, *E. coli*-induced hypothermia was most severe in *E. coli*-only challenged animals, which correlated with an increased peritoneal bacterial load (Figure 2A,B (Fig. 2); r_s_=–0.7074; p<0.0001; n=74). *L. sigmodontis* infection further resulted in an increased total number of peritoneal macrophages following *E. coli* challenge (Figure 2C (Fig. 2)). Moreover, peritoneal macrophages of *L. sigmodontis*-infected animals had an increased number and expression of RELMα and a slightly reduced expression of the activation marker CD86 following *E. coli* injection, indicating the induction of alternatively activated macrophages (Figure 2D–F (Fig. 2)). Depletion of TGFβ did not reverse these *L. sigmodontis*-induced changes in macrophage phenotype and numbers and had no impact on macrophage numbers and frequency and RELMα expression in *E. coli*-only challenged mice. 

### TGFβ depletion does neither increase inflammation nor change numbers of monocytes, neutrophils and eosinophils during SIRS 

**Figure 3:**
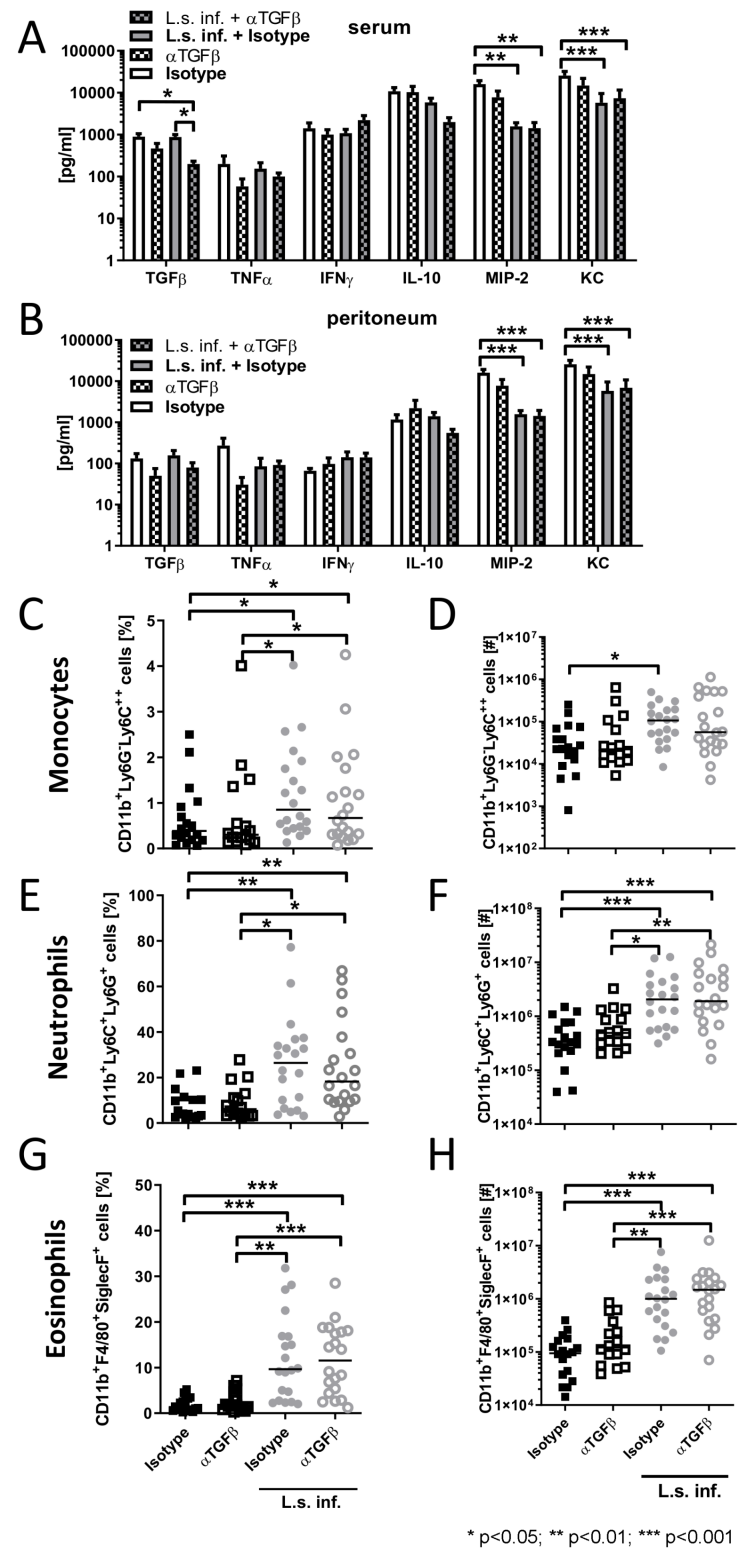
TGFβ depletion does not alter pro-inflammatory cytokine/chemokine production, peritoneal composition of monocytes, neutrophils and eosinophils during SIRS and their expansion in *L. sigmodontis*-infected mice. (A) Serum and (B) peritoneal cytokine/chemokine levels six hours after *E. coli* injection. (C,D) Relative and absolute number of peritoneal CD11b^+^Ly6G^–^Ly6C^++^ monocytes, (E,F) CD11b^+^Ly6G^+^Ly6C^+^ neutrophils and CD11b^+^F4/80^med^SiglecF^+^ eosinophils (G,H) six hours after *E. coli* injection in mice that were infected for 90 days with *L. sigmodontis* and uninfected controls and received either anti-TGFβ or an isotype control before *E. coli* challenge. Data represent three pooled independent experiments with at least six mice per group. Data are shown as mean +SEM (A,B) and median (C–H) and was tested for statistical significance by Kruskal-Wallis test and Dunn’s Multiple Comparison post hoc test.

To examine the role of TGFβ on systemic and local inflammation, pro-inflammatory and anti-inflammatory cytokines and chemokines were analyzed in the peritoneum as well as in the blood 6 h after *E. coli* injection in *L.* *sigmodontis*-infected animals and controls. TGFβ depletion reduced TGFβ concentrations in serum and peritoneum, reaching statistical significance for the comparison in serum between anti-TGFβ treated, *L. sigmodontis*-infected mice and both isotype treated controls (Figure 3A,B (Fig. 3)). *L. sigmodontis* infection significantly reduced KC and MIP-2 levels following *E. coli* challenge (Figure 3A,B (Fig. 3)). However, TGFβ depletion had no impact on KC and MIP2 levels, independent on the presence of *L. sigmodontis* infection. Similarly, depletion of TGFβ did not alter IFNγ concentrations in the serum and peritoneum of *L. sigmodontis*-infected mice (Figure 3A,B (Fig. 3)). In contrast, TGFβ depletion tended to reduce TNF concentrations in blood and peritoneum of *E. coli*-only challenged controls (Figure 3A,B (Fig. 3)). 

Since TGFβ is a potent chemoattractant and activator for neutrophils [14] and neutrophil influx to the site of infection correlated with an improved sepsis outcome [15], we investigated whether TGFβ depletion alters the peritoneal composition following *E. coli* challenge. Six hours after *E. coli* injection, eosinophil, neutrophil and monocyte frequencies and total numbers were significantly increased in *L. sigmodontis*-infected animals compared to *E. coli*-only challenged controls (Figure 3C–H (Fig. 3)). Depletion of TGFβ had no impact on the peritoneal cellular composition of these cell types following *E. coli* challenge, independent of the infection status with *L. sigmodontis*. Thus, our data indicate that TGFβ has no crucial role in the recruitment and maintenance of neutrophils, eosinophils and monocytes during the acute phase of sepsis.

## Discussion

In this study, we examined the effect of TGFβ removal in *L. sigmodontis*-infected and uninfected BALB/c mice 6 h after *E. coli* injection. The *L. sigmodontis* infection resulted in a milder *E. coli*-induced hypothermia and a reduced bacterial load. Depletion of TGFβ did hereby neither aggravate *E. coli*-induced hypothermia nor increase the peritoneal bacterial load in *L. sigmodontis*-infected nor uninfected animals. These results confirm our previous findings that chronic filarial infection improves bacterial clearance and ameliorates *E. coli*-induced hypothermia [10], suggesting that the *L. sigmodontis*-mediated protective effect is independent on TGFβ. In addition, the expression of RELMα and CD86 on peritoneal macrophages was not altered by TGFβ depletion, indicating that the *L. sigmodontis*-induced macrophage modulation is not due to TGFβ during acute sepsis. Moreover, eosinopenia as a controversially discussed marker for sepsis severity [16], [17] and monocytes as potent modulators of inflammation during the SIRS phase and initiators of the subsequent immunosuppressive phase [18] were increased together with neutrophils after *E. coli* challenge in filariae-infected mice, independent on TGFβ removal. Interestingly, despite TGFβ depletion, levels of pro-inflammatory mediators, bacterial burden and hypothermia remained improved in chronic *L. sigmodontis*-infected mice. Thus, our data indicates that lack of TGFβ signaling does not substantially increase local and systemic pro-inflammatory cytokine and chemokine concentrations during SIRS. 

## Conclusions

Our study suggests that TGFβ is not essentially involved in the immune response during the early phase of an *E. coli*-induced sepsis. Peritoneal bacterial load, *E. coli*-induced hypothermia, systemic and local inflammation, as well as the cellular composition within the peritoneum were not altered by TGFβ depletion before the *E. coli* challenge. Furthermore, the *L. sigmodontis*-mediated protective effect against an *E. coli*-induced sepsis was still present in TGFβ-depleted mice. Future studies should therefore investigate whether other regulatory components like IL-10 or PD1/PDL1 interactions compensate for the lack of TGFβ signaling and whether TGFβ is essential during the CARS phase of sepsis.

## Notes

### Competing interests

The authors declare that they have no competing interests.

### Acknowledgments

We thank Anna-Lena Neumann for her assistance in performing the experiments and Prof. Dr. Lina Gölz for critical reading of the manuscript. 

### Funding

BB was supported by the Jürgen Manchot Stiftung, Düsseldorf. AH is member of the Excellence Cluster Immunosensation (DFG, EXC1023). This work was funded by the German Research Foundation (HU 2144/1-1); intramural funding by the University Hospital of Bonn (BONFOR, 2010-1-16 and 2011-1-10); and the People Programme (Marie Curie Actions) of the European Union’s Seventh Framework Programme FP7/2007–2013 under Research Executive Agency Grant GA 276704. 

## References

[1] Borish LC, Steinke JW (2003). 2. Cytokines and chemokines. J Allergy Clin Immunol.

[2] Akhurst RJ, Hata A (2012). Targeting the TGFβ signalling pathway in disease. Nat Rev Drug Discov.

[3] Hübner MP, Shi Y, Torrero MN, Mueller E, Larson D, Soloviova K, Gondorf F, Hoerauf A, Killoran KE, Stocker JT, Davies SJ, Tarbell KV, Mitre E (2012). Helminth protection against autoimmune diabetes in nonobese diabetic mice is independent of a type 2 immune shift and requires TGF-β. J Immunol.

[4] Dittrich AM, Erbacher A, Specht S, Diesner F, Krokowski M, Avagyan A, Stock P, Ahrens B, Hoffmann WH, Hoerauf A, Hamelmann E (2008). Helminth infection with Litomosoides sigmodontis induces regulatory T cells and inhibits allergic sensitization, airway inflammation, and hyperreactivity in a murine asthma model. J Immunol.

[5] Ahmad S, Choudhry MA, Shankar R, Sayeed MM (1997). Transforming growth factor-beta negatively modulates T-cell responses in sepsis. FEBS Lett.

[6] Bae JS, Lee W, Nam JO, Kim JE, Kim SW, Kim IS (2014). Transforming growth factor β-induced protein promotes severe vascular inflammatory responses. Am J Respir Crit Care Med.

[7] Weehuizen TA, Wieland CW, van der Windt GJ, Duitman JW, Boon L, Day NP, Peacock SJ, van der Poll T, Wiersinga WJ (2012). Expression and function of transforming growth factor β in melioidosis. Infect Immun.

[8] McCartney-Francis N, Jin W, Wahl SM (2004). Aberrant Toll receptor expression and endotoxin hypersensitivity in mice lacking a functional TGF-beta 1 signaling pathway. J Immunol.

[9] Pender BS, Chen H, Ashton S, Wise WC, Zingarelli B, Cusumano V, Cook JA (1996). Transforming growth factor beta 1 alters rat peritoneal macrophage mediator production and improves survival during endotoxic shock. Eur Cytokine Netw.

[10] Gondorf F, Berbudi A, Buerfent BC, Ajendra J, Bloemker D, Specht S, Schmidt D, Neumann AL, Layland LE, Hoerauf A, Hübner MP (2015). Chronic filarial infection provides protection against bacterial sepsis by functionally reprogramming macrophages. PLoS Pathog.

[11] Buerfent BC, Gondorf F, Wohlleber D, Schumak B, Hoerauf A, Hübner MP (2015). Escherichia coli-induced immune paralysis is not exacerbated during chronic filarial infection. Immunology.

[12] Buerfent BC, Gölz L, Hofmann A, Rühl H, Stamminger W, Fricker N, Hess T, Oldenburg J, Nöthen MM, Schumacher J, Hübner MP, Hoerauf A (2019). Transcriptome-wide analysis of filarial extract-primed human monocytes reveal changes in LPS-induced PTX3 expression levels. Sci Rep.

[13] Ajendra J, Specht S, Neumann AL, Gondorf F, Schmidt D, Gentil K, Hoffmann WH, Taylor MJ, Hoerauf A, Hübner MP (2014). ST2 deficiency does not impair type 2 immune responses during chronic filarial infection but leads to an increased microfilaremia due to an impaired splenic microfilarial clearance. PLoS ONE.

[14] Brandes ME, Mai UE, Ohura K, Wahl SM (1991). Type I transforming growth factor-beta receptors on neutrophils mediate chemotaxis to transforming growth factor-beta. J Immunol.

[15] Alves-Filho JC, Sônego F, Souto FO, Freitas A, Verri WA, Auxiliadora-Martins M, Basile-Filho A, McKenzie AN, Xu D, Cunha FQ, Liew FY (2010). Interleukin-33 attenuates sepsis by enhancing neutrophil influx to the site of infection. Nat Med.

[16] Merino CA, Martínez FT, Cardemil F, Rodríguez JR (2012). Absolute eosinophils count as a marker of mortality in patients with severe sepsis and septic shock in an intensive care unit. J Crit Care.

[17] Escobar-Valdivia EJ, González-Aguirre JE, Carrillo-Cisneros ER, Guerra-Leza KC, Mercado-Longoría R (2015). Eosinophil count at intensive care unit admission was not predictor of hospital mortality: results of a case control study. J Intensive Care.

[18] Shalova IN, Lim JY, Chittezhath M, Zinkernagel AS, Beasley F, Hernández-Jiménez E, Toledano V, Cubillos-Zapata C, Rapisarda A, Chen J, Duan K, Yang H, Poidinger M, Melillo G, Nizet V, Arnalich F, López-Collazo E, Biswas SK (2015). Human monocytes undergo functional re-programming during sepsis mediated by hypoxia-inducible factor-1α. Immunity.

